# Lessons for cross-species viral transmission surveillance from highly pathogenic avian influenza Korean cat shelter outbreaks

**DOI:** 10.1038/s41467-023-42738-w

**Published:** 2023-10-31

**Authors:** Younjung Kim, Guillaume Fournié, Raphaëlle Métras, Daesub Song, Christl A. Donnelly, Dirk U. Pfeiffer, Pierre Nouvellet

**Affiliations:** 1 Sorbonne Université, INSERM, Institut Pierre Louis d’Épidémiologie et de Santé Publique (IPLESP), UMRS 1136 Paris, France; 2 Department of Ecology and Evolution, School of Life Sciences, University of Sussex, Brighton and Hove, UK; 3 Department of Pathobiology and Population Sciences, Royal Veterinary College, London, UK; 4 Université de Lyon, INRAE, VetAgro Sup, UMR EPIA, Marcy l′Etoile, France; 5 Université Clermont Auvergne, INRAE, VetAgro Sup, UMR EPIA, Saint-Gènes-Champanelle, France; 6 Department of Veterinary Medicine Virology Laboratory, College of Veterinary Medicine and Research Institute for Veterinary Science, Seoul National University, Seoul, Republic of Korea; 7 Department of Statistics, University of Oxford, Oxford, UK; 8 Pandemic Sciences Institute, University of Oxford, Oxford, UK; 9 MRC Centre for Global Infectious Disease Analysis and Abdul Latif Jameel Institute for Disease and Emergency Analytics, Imperial College London, London, UK; 10 City University of Hong Kong, Hong Kong SAR, China

**Keywords:** Viral infection, Influenza virus, Epidemiology

## Abstract

In this Comment, the authors describe recent outbreaks of highly pathogenic avian influenza in cat shelters in Seoul, South Korea. They discuss potential routes of transmission and describe implications for surveillance of spillover infections in animals in non-agricultural settings.

## Teaser

Recent highly pathogenic avian influenza A(H5N1) outbreaks in two Korean cat shelters highlight the need to enhance surveillance for cross-species viral transmission into animal populations kept by humans for non-agricultural or non-conventional livestock farming purposes from a One Health perspective.

Cross-species viral transmission refers to the infection of a new host species by a virus, commonly referred to as ‘spillover'^1,2^. The current COVID-19 pandemic has clearly illustrated how cross-species viral transmission can negatively impact both human and animal health, with the potential to trigger future pandemics, thereby highlighting the importance of having effective and efficient surveillance for cross-species viral transmission in place. Nonetheless, the challenge always remains in identifying the interfaces for cross-species viral transmission and distributing surveillance efforts optimally amongst these interfaces.

The research and public attention on cross-species viral transmission has primarily focused on enhancing our knowledge about a pool of potential zoonotic viruses in wildlife, monitoring the prevalence of known zoonotic viruses within wildlife or livestock, and assessing the risk of their transmission to humans, along with syndromic surveillance targeted at unexplained clinical cases in livestock and humans. Above all, these surveillance activities are concentrated at the interfaces where the economic and health impacts of cross-species transmission are considered significant^1,3,4^. However, surveillance in animal populations raised for non-conventional production purposes (e.g. minks and foxes for fur production) and those in non-agricultural settings (e.g. animal shelters, zoos, and places where visitors can touch companion, or wild animals) remains scarce globally, despite increasing attention to surveillance on mink and fox farms^5^ in the context of recent SARS-CoV-2 and highly pathogenic avian influenza (HPAI) A(H5N1) outbreaks in these settings^6–9^.

On July 25^th^ and 31^st^, 2023, highly pathogenic avian influenza A(H5N1) infection, which has the potential to cause fatal disease in humans^10^, was confirmed in two cat shelters in Seoul, South Korea^11–13^. Briefly, at the cat shelter where the infection was first confirmed (Shelter 1), 38 out of the 40 cats died within a month, with the time between two consecutive deaths being ≤2 days^13^. This surge in deaths prompted the owner of Shelter 1 and its veterinarian to suspect a cause other than common respiratory infections in cats. Samples from two of the cats that had died from suspected respiratory infections were sent to a private diagnostic laboratory and confirmed to contain HPAI A(H5N1) virus by the Laboratory of Virology at the College of Veterinary Medicine, Seoul National University^13^. Then, the infection was confirmed again by the Animal and Plant Quarantine Agency (APQA)^11^ after it was formally reported to the agency according to the Act on the Prevention of Animal Contagious Diseases: the Act mandates reporting animals, including cats, that have died of unidentified diseases or are suspected or confirmed to have been infected by contagious animal diseases types I, II, and III as defined by the Act^14^. Several days later, a veterinarian reported respiratory symptoms in a cat from another shelter in Seoul (Shelter 2), and HPAI A(H5N1) infection was subsequently confirmed by the APQA^12^. As of October 2023, influenza A(H5N1) infection has been confirmed in five cats from Shelter 1 and four cats from Shelter 2, all of which were reported to have died^15,16^. The information on the number of cats with negative test results has not been released yet.

Avian influenza surveillance in South Korea is primarily focused on the detection of infections on poultry farms and in wild birds. Surveillance in mammals started early this year. However, it only focused on specific wild mammalian species and did not include domestic cats^17^. Not surprisingly, the detection of the virus in cats in both these cat shelters within a short timespan attracted widespread attention. A subsequent epidemiological investigation by the Ministry of Agriculture, Food and Rural Affairs (MAFRA) detected influenza A(H5N1) virus in cat food collected in Shelter 2, which contained duck meat, but not in cat food containing chicken meat. The MAFRA investigation concluded that raw duck and chicken meat had not been properly processed by the company that produced the cat food found to be contaminated^15,18^. In a swift response, the MAFRA ordered the recall of all cat food production batches containing duck or chicken meat that were deemed at risk. Approximately 13,200 units of the two recalled products had already been sold to 286 customers, but no signs of infection have been reported amongst their cats which were monitored twice at a 14-day interval^15^. Considering also that all cats in the two shelters were known to have stayed indoors and HPAI infection had been confirmed on the country’s poultry farms including duck farms until April 2023^16^, it is plausible that influenza A(H5N1) viral incursion originated from contaminated duck meat contained in cat food, particularly in Shelter 2. However, as of this writing, no definitive epidemiological or genomic evidence has been released by the MAFRA, and therefore, it is unknown whether cats in Shelter 1 were fed the same or similar products and whether cat-to-cat transmission occurred following initial exposure in the two shelters and, if it did, what its relative contribution was compared to potential food-to-cat transmission.

So far, the management of the influenza A(H5N1) outbreaks in the cat shelters can be considered an example of good practice of surveillance and epidemiological investigations related to cross-species viral transmission. It has successfully detected influenza A(H5N1) infection in cats, identified a potential source of infection in at least one of the two shelters, and resulted in prompt transmission control measures. However, the question arises whether the MAFRA and APQA would be able to respond as successfully to cross-species viral transmission occurring in other epidemiological contexts. It has to be emphasised that the detection of influenza A(H5N1) outbreaks in the two cat shelters was initiated by the sudden deaths of multiple cats in Shelter 1 noticed by its owner and veterinarian. Subsequent steps taken by the veterinarians and virologists then led to the notification of the authorities, thereby initiating more detailed epidemiological investigations. This underscores the potential challenge of detecting cross-species influenza A(H5N1) infection and identifying a potential source of contagion if contaminated cat food was solely distributed to privately-owned cats rather than shelter cats, or if any element of the aforementioned chain of events was absent. There are several possible reasons for this:

Firstly, under a given level of viral pressure, stochastic viral extinction may be more likely to occur when only a few cats in individual households are exposed to the virus as opposed to a larger group of cats. This is due to the increased contact rates between cats in a shelter as well as the increased likelihood of the virus successfully adapting to cats and subsequently transmitting between them, especially considering immunological barriers within individual cats^1^, although it is currently unknown whether any cat-to-cat transmission had occurred in the two shelters.

Secondly, the presence of infection of cats in different households would be more likely to be missed by animal health authorities. This is due to the challenge that veterinarians would face in suspecting influenza A(H5N1) infection when individual cats are presented to different veterinary clinics. Although HPAI infection in cats is notifiable, it is much less common than other respiratory infections in cats^19^ and is difficult to distinguish during the early infection stages. The fact that the reporting from Shelter 1 was made only after a substantial number of cats had died further emphasises the challenge veterinarians would face with limited prior experiences and knowledge about the risk and clinical manifestation of HPAI infection in cats. The recent identification of influenza A(H5N1) infections in privately-owned cats in Poland demonstrates that detecting the infection in such a scenario is not entirely implausible^20^. However, it also indicates that undetected exposure of privately-owned cats to the virus could be frequent, consistent with serological evidence of avian influenza A infections in cats from different countries including household and shelter cats in South Korea^21,22^. Finally, if the virus is spread to cats through other routes (e.g. hunting and ingestion of infected birds by stray or privately-owned cats with outdoor access), the ability to establish plausible and specific epidemiological links would be challenged by the difficulty of conducting timely and targeted sampling as well as comprehensive history taking. While inadequately processed uncooked poultry meat leftovers in Shelter 2 were successfully collected and tested, it remains uncertain if the same could be achieved in other scenarios. This is exemplified by the recent influenza A(H5N1) infections of cats from individual households in Poland, where purchased food could only be tested for suspected cases, but not for confirmed cases^20^.

The influenza A(H5N1) outbreaks in the cat shelters in South Korea, along with an increasing number of reports of influenza A(H5N1) infection in mammals^5,7,20^, suggest the need to enhance the effectiveness of infectious disease surveillance and prevention in any settings where a group of potentially susceptible animals are kept in close contact. This is particularly important for groups of animals that typically are not covered by regulatory efforts aimed at maintaining or improving animal health and welfare standards. These animal groups could not only provide environments for cross-species viral transmission, promoting zoonotic risk, but could also serve as sentinels for detecting such events, thereby offering opportunities to assess and then mitigate such risk. Several strategies can be implemented:

Firstly, it is important to require the aforementioned settings to record adverse animal health events at individual (e.g. clinical signs) and group levels (e.g. baseline mortality and morbidity), along with environmental factors (e.g. access to/from wildlife, and introduction of new animals) and husbandry conditions (e.g. food and water source), in systematic ways. In the event of cross-species viral transmission, this information will help to better understand the source of infection and thus inform the implementation of preventive and control measures. Such a recording practice appears to have been lacking in these particular settings, very much in contrast to conventional livestock farming where the recording is routinely practised.

Secondly, regularly training staff in the aforementioned settings to report any unusual clinical signs or trends is crucial for timely and sensitive early detection, coupled with the above recording practice. For this, animal health authorities should define clear reporting criteria, ensure their staff’s familiarity with these criteria, and work to minimise the risk of underreporting due to potential stigmatisation. Additionally, animals in these settings need to be regularly examined by veterinarians to aid the timely recognition of unusual health events, which are often neglected due to economic constraints. Importantly, animal health authorities could play a role by designating veterinary staff to provide regular trainings to the staff about the recording practice and reporting criteria and to examine the animals.

Thirdly, the identification of inadequately processed uncooked poultry meat as a potential common source of influenza A(H5N1) infection in cats in both South Korea and Poland provides strong justification for prohibiting or limiting feeding of uncooked meat to animals kept for non-conventional production purposes and those in non-agricultural settings, in order to minimise the risk of cross-species transmission of influenza A(H5N1) virus and other viruses. Given the widespread availability of heat-treated commercial pet foods and the option to cook raw meat before feeding, this recommendation might be feasible for most animal species in these settings. Furthermore, if necessary, a ban on feeding uncooked meat to certain animal species could be implemented where cross-species transmission has been already reported or the risk is considered high (e.g. uncooked poultry meat to cats, minks, and foxes), similar to the bans on swill feeding in many countries to prevent the introduction of African swine fever virus into domestic pig farms^23^. Finally, there is a need for continued discussion on how to implement measures to prevent and control cross-species viral transmission. It is crucial to involve all relevant stakeholders in this discussion, particularly the staff of the aforementioned animal settings, as their understanding of the implications of animal keeping for animal and human health might be less than amongst those involved in conventional livestock farming.

In conclusion, the recent influenza A(H5N1) outbreaks in South Korean cat shelters illustrate the risk of cross-species viral transmission in animals kept for non-agricultural or non-conventional livestock farming purposes, considered to present potential threats to human health. These events emphasise the need to strengthen surveillance of cross-species viral transmission events within these specific animal populations. However, it is also important to acknowledge that such efforts would require supplementary resources for national animal disease surveillance systems. While the scale of these additional resources may be relatively modest compared to those already invested in conventional livestock health surveillance, it could still pose a significant economic burden, especially in regions where the animal disease surveillance system is already poorly resourced. Therefore, it will be crucial to set realistic priorities by conducting risk assessments on the interfaces at which cross-species transmission has already been detected or expected to occur, in order to allocate limited surveillance resources based on the outcome of these risk assessments. By adopting such approaches, we can better prepare to protect against future zoonotic threats, thereby safeguarding both human and animal populations globally.
